# The role of cardiac transcription factor *NKX2-5* in regulating the human cardiac miRNAome

**DOI:** 10.1038/s41598-019-52280-9

**Published:** 2019-11-04

**Authors:** Deevina Arasaratnam, Katrina M. Bell, Choon Boon Sim, Kathy Koutsis, David J. Anderson, Elizabeth L. Qian, Edouard G. Stanley, Andrew G. Elefanty, Michael M. Cheung, Alicia Oshlack, Anthony J. White, Charbel Abi Khalil, James E. Hudson, Enzo R. Porrello, David A. Elliott

**Affiliations:** 1 Murdoch Children’s Research Institute, Royal Children’s Hospital, Flemington Road, Parkville, Victoria 3052 Australia; 2 Australian Regenerative Medicine Institute, Monash University, Clayton, Victoria 3800 Australia; 3 Department of Pediatrics, The Royal Children’s Hospital, University of Melbourne, Parkville, Victoria 3052 Australia; 4 Department of Anatomy and Developmental Biology, Monash University, Clayton, Victoria 3800 Australia; 5 Monash Heart, Monash Medical Centre, Monash University, Clayton, Victoria 3800 Australia; 6 Department of Genetic Medicine and Medicine, Weill Cornell Medical College-Qatar, Doha, Qatar; 7 QIMR Berghofer Medical Research Institute, Herston, Queensland 4006 Australia; 8 Department of Physiology, School of Biomedical Sciences, The University of Melbourne, Parkville, Victoria 3010 Australia

**Keywords:** Differentiation, RNA

## Abstract

MicroRNAs (miRNAs) are translational regulatory molecules with recognised roles in heart development and disease. Therefore, it is important to define the human miRNA expression profile in cardiac progenitors and early-differentiated cardiomyocytes and to determine whether critical cardiac transcription factors such as *NKX2-5* regulate miRNA expression. We used an *NKX2-5*^*eGFP/w*^ reporter line to isolate both cardiac committed mesoderm and cardiomyocytes. We identified 11 miRNAs that were differentially expressed in *NKX2-5* -expressing cardiac mesoderm compared to non-cardiac mesoderm. Subsequent profiling revealed that the canonical myogenic miRNAs including *MIR1-1*, *MIR133A1* and *MIR208A* were enriched in cardiomyocytes. Strikingly, deletion of *NKX2-5* did not result in gross changes in the cardiac miRNA profile, either at committed mesoderm or cardiomyocyte stages. Thus, in early human cardiomyocyte commitment and differentiation, the cardiac myogenic miRNA program is predominantly regulated independently of the highly conserved *NKX2-5* -dependant gene regulatory network.

## Introduction

The heart is the first functional organ to develop in the human embryo, and the organ most commonly affected by disease in infants and adults. Heart development is tightly controlled by an evolutionarily conserved network of transcription factors and disruption of this network can result in a variety of congenital heart malformations. MicroRNAs (miRNAs), short (19–22 base pair) RNA regulatory molecules, add another layer of regulatory precision to reinforce core cardiac transcriptional networks^1^. Furthermore, the cardiogenic regulatory framework is reinforced by the direct control of certain microRNAs by critical myogenic transcription factors, including serum response factor (SRF), myocyte enhancer factor-2 (MEF2c) and GATA4^2–4^. In addition to their key roles in heart development, miRNAs are critical to maintaining cardiac tissue homeostasis and function^1,5^. For example, global perturbation of the cardiac miRNAome via deletion of the microRNA processing enzymes Dicer and *DGCR8* leads to dilated cardiomyopathy^6–8^. In view of this, miRNAs emerged as a new class of functional regulators of cardiomyogenesis and heart disease^1,9–12^. Given their important role in both heart development and function, careful compellation of the human cardiomyocyte miRNAome will facilitate studies in a number of areas in cardiac biology. Human pluripotent stem cells offer an opportunity to study the expression profile of miRNAs in human heart muscle cells^13–17^.

Here, we have used a well-established *NKX2-5* cardiac reporter line^18^ and loss-of-function model^19^ to determine whether the human cardiac miRNAome is directly dependent on NKX2-5, a transcriptional factor that is essential for mammalian heart development^19–21^. We show that the cardiomyogenic miRNA program is activated early during human cardiomyocyte differentiation *in vitro*. The expression of the myogenic miRNAs^9^, *MIR1-1*, *MIR133A1*, *MIR208A* and *MIR499A*, was established by day 10 of pluripotent stem cell differentiation into the cardiac lineage. However, *NKX2-5* was dispensable for maintenance of the human cardiomyocyte miRNAome, with no differentially expressed miRNAs identified in either cardiac committed mesodermal progenitors or immature differentiated cardiomyocytes. These data suggest that, in the main, establishing the cardiomyogenic miRNA program occurs independently of the highly conserved core NKX2-5 gene regulatory network.

## Results

### Defining miRNA expression profiles in hESC derived human cardiac progenitors

**Figure 1:**
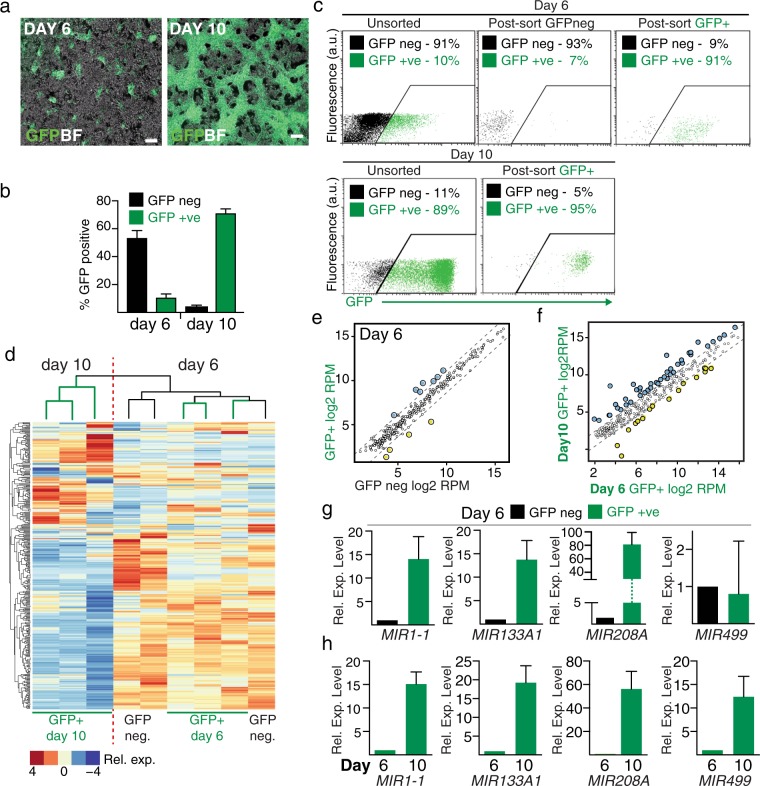
MicroRNA expression profiling of *NKX2-5* positive cardiac progenitors and cardiomyocytes. (**a)** Epifluorescent images of differentiating *NKX2-5*^*eGFP/w*^ cells (Scale bar, 100 μm). (**b**) Percentages of GFP positive and negative cells at day 6 and 10 of differentiation. Data shows mean ± SEM (n = 3) determined by flow cytometry. (**c**) Flow cytometric purification of cardiac progenitors and cardiomyocytes based on eGFP expression from the *NKX2-5* locus. Percentage of cells are indicated on plots. (**d**) Heat map of unsupervised hierarchical clustering of microRNA sequence profile showing that samples cluster according to day of differentiation (log_2_ RPM values). (**e)** Dot plot representation of miRNA-seq absolute expression (log_2_ RPM values) for miRNAs at day 6 of differentiation. Dashed lines mark 2-fold differential expression level. (**f)** Dot plot representation of miRNA-seq absolute expression (log_2_ RPM values) comparing day 6 and day 10 GFP positive cells. Dashed lines mark 2-fold differential expression level. (**g**) Q-PCR profiling supports miRNA sequencing data and confirms upregulation of canonical myogenic miRNAs in GFP^+^ cells at day 6. (**h)** Q-PCR of myomiRs demonstrates they are more highly expressed in day 10 cardiomyocytes than day 6 GFP^+^ cells.

We utilized the *NKX2-5*^*eGFP/w*^ hESC reporter cell line, in which enhanced GFP (eGFP) is expressed from the *NKX2–5* locus^18^, to isolate hESC-derived cardiac mesodermal progenitors and cardiomyocytes by flow cytometry. Using a monolayer differentiation protocol^22,23^ GFP^+^ myogenic-lineages emerged between days 6 to 14 of the differentiation (Fig. 1a). To obtain a profile of miRNAs during early cardiomyocyte differentiation, cells were harvested and sorted into three populations: *NKX2-5* negative mesoderm (day 6 GFP^neg^ cells), cardiac mesodermal progenitors (day 6 GFP^+^ cells) and early cardiomyocytes (day 10 GFP^+^ cells) for sequencing of small RNA species (Fig. 1b,c). A summary of small RNA sequencing data is shown in Supplementary Table 1. The average number of high-quality reads in the day 6 GFP^neg^, day 6 GFP^+^ and day 10 GFP^+^ populations were 2.01 ± 0.10 × 10^6^ (n = 3), 1.71 ± 0.08 × 10^6^ (n = 3), and (2.35 ± 0.08) x 10^6^ (n = 3), respectively. Small nucleolar RNAs (snoRNAs) were also detected in day 6 GFP^+^ and GFP^neg^ populations (1.18 ± 0.24 × 10^5^ (n = 3) and 3.19 ± 1.7 × 10^5^(n = 3),reads respectively) and day 10 cardiomyocytes (1.6 ± 0.17 × 10^5^ (n = 3)). Thus, other small non-coding RNA populations such as snoRNAs are also present during human cardiac differentiation, but their functions are currently unclear.

Unsupervised hierarchical clustering indicated that the miRNA profiles of day 6 GFP^+^ (cardiac mesoderm progenitors) and GFP^neg^ populations were more closely related to each other than to day 10 cardiomyocytes (Fig. 1d and Supplementary Table 2). The similarity of miRNA expression profiles between the *NKX2-5* positive and negative populations at day 6 most likely reflects the recent emergence of the *NKX2-5*^+^ cells from the pool of mesodermal cells. Differential expression analysis between day 6 GFP^+^ and GFP^neg^ cells identified seven miRNAs enriched in the GFP^+^ population (Fold change >2 with adjusted p-value < 0.05)(Fig. 1e; Supplementary Table 2). Among the seven overrepresented miRNAs, six have been reported to have important roles in murine cardiac development. For example, *MIR133A1* and *MIR133A2* play critical roles in promoting pre-cardiac mesoderm, while suppressing endodermal and neuroectodermal lineages^24^. We also observed higher levels of *MIR125B1* in d6 cardiac progenitors, which plays a role in mesoderm development^25^ and regulates *MEF2d* in the HL-1 atrial myocyte cell line. Q-PCR of the cardiomyogenic miRNAs *MIR1-1*, *MIR133A*1 and *MIR208A* showed these were more highly expressed in day 6 cardiac mesoderm while MIR499 was not differentially expressed between GFP^neg^ and GFP^+^ cells (Fig. 1g,h). In addition, four miRNAs (*MIR122*, *MIR126*, *MIR1247* and *MIR127*) were expressed at higher levels in GFP^neg^ mesodermal progenitors than GFP^+^ cells. (Fig. 1e; Supplementary Table 2). These miRNAs have been implicated in a wide range of non-cardiac processes such as liver homeostasis, hematopoiesis and self-renewal of cancer stem cells^26–28^.

To identify miRNAs that may be important drivers of cardiomyocyte differentiation, we compared the expression profile of d6 and d10 GFP positive cells (Fig. 1d,f). This analysis identified a total of 112 differentially expressed miRNAs (Supplementary Table 2). Studies in the mouse have identified a core miRNA network involved in cardiomyocyte function^9^. The known myomiRs (*miRNA-1-1,-1-2,133b* and *-208*)^9^ were all expressed at higher levels at day 10 compared to day 6. Moreover, *MIR499*, a miRNA located within the intron of *myosin heavy chain 7b* (*MYH7b*), which encodes a cardiomyocyte sarcomeric protein, showed the highest differential expression (5.62 fold) in day 10 cardiomyocytes^29^. Q-PCR profiling of miRNA expression in day 10 GFP^+^ cardiomyocytes confirmed these key miRNAs are more highly expressed in cells committed to the cardiomyocyte lineage (Fig. 1h). *MIR1-1*, *MIR133A1*, *MIR208A* and *MIR145* expression levels are further upregulated in the day 10 *NKX2-5*^*eGFP/w*^ GFP^+^ populations relative to day 6 populations. The data indicates that expression of canonical myogenic miRNAs is established early in cardiomyocyte differentiation.

A set of miRNAs that regulate DNA synthesis were more highly expressed in the mesoderm progenitor stage compared to day 10 cardiomyocytes (Supplementary Fig. 1). The Hippo-YAP pathway is an established driver of cardiomyocyte proliferation^30–32^ and miRNA regulators of this network are differentially expressed between day 6 mesodermal progenitors and the early cardiomyocyte cells at day 10 (Supplementary Fig. 2). For example, *MIR30E*, which is a known repressor of YAP1 expression^33^ is more highly (2 fold) expressed in day 10 cardiomyocytes. Conversely, *MIR302a* (2.2 fold) and *MIR302e* (2.5 fold) are found in the highly proliferative cardiac progenitor population but are lower in day 10 cardiomyocytes. This finding is consistent with the known function of the *miR302–367* cluster in repressing the Hippo pathway components *Mst1*, *Lats3* and *Mob1b* and, therefore, cardiomyocyte proliferation^31^. Thus, the *NKX2-5*^*eGFP*^ positive day 6 population expresses a cohort of miRNAs consistent with the highly proliferative nature of this cardiac progenitor population.

### *NKX2-5* is not required to establish the cardiac microRNAome

**Figure 2:**
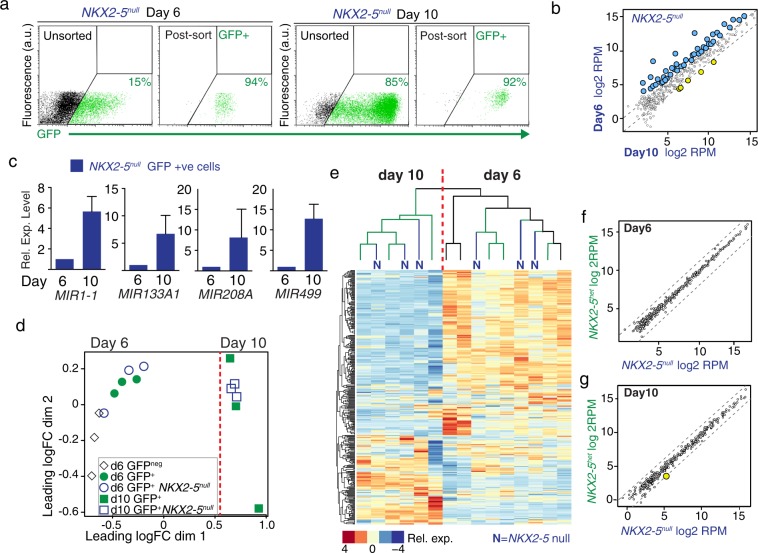
*NKX2-5* is dispensable for establishing the appropriate human cardiomyocyte microRNA profile. (**a**) Flow cytometric purification of *NKX2-5* null (*NKX2-5*^*−/−*^) cardiac progenitors and cardiomyocytes based on eGFP expression from the *NKX2-5* locus. Percentage of GFP^+^ cells shown on plots. (**b**) Dot plot representation of miRNA-seq expression (log_2_ RPKM values) for miRNAs in *NKX2-5*^*−/−*^ GFP positive cells at day 6 and day 10 of differentiation. Dashed lines mark 2-fold differential expression level. (**c**) Q-PCR profiling supports miRNA sequencing data and shows upregulation of canonical myogenic miRNAs during differentiation in *NKX2-5* null cells. (**d)** Multidimensional scaling plot of miRNA expression profiles from differentiating *NKX2-5* heterozyogote cells (GFP^neg^, GFP^+^) and *NKX2-5* null (d6 or d10 GFP^+^ *NKX2-5*^null^) at day 6 and 10 of differentiation. (**e**) Heat map of unsupervised hierarchical clustering of microRNA sequence profile (log_2_ RPM values); (**d**,**e)** show that samples cluster according to day of differentiation and not *NKX2-5* genotype. (**f)** Scatter plot of miRNA-seq expression (log_2_ RPM values) for miRNAs at day 6 of differentiation from *NKX2-5eGFP/* + and *NKX2-5−/−* cells showing no significantly differential expressed miRNAs. (**g)** Scatter plot of miRNA-seq absolute expression (log_2_ RPM values) for miRNAs at day 10 of differentiation from *NKX2-5eGFP/* + and *NKX2-5−/−* cardiomyocytes showing that no miRNAs are *NKX2-5* dependent. Yellow dot denotes *MIR138-2*.

In order to determine if *NKX2-5* is required to establish the human cardiac miRNAome, we isolated cardiac progenitors and early cardiomyocytes from *NKX2-5*^*−/−*^ (*NKX2-5*^*eGFP/eGFP*^) hESCs^19^. Day 6 cardiac mesodermal cells and day 10 cardiomyocytes were isolated by flow cytometry and subjected to small RNA sequencing (Fig. 2a,b). Similar to the heterozygous *NKX2-5*^*eGFP/w*^ cells, the day 10 *NKX2-5*^*−/−*^ cells differentially expressed miRNAs, including members of the myomiRs consistent with the cardiomyogenic cell phenotype (Fig. 2b; Supplementary Table 3). This result was confirmed by Q-PCR for the myomiRs *MIR1-1*, *MIR133A1*, *MIR208A* and *MIR499*. Strikingly, non-hierarchical clustering and principal component analysis demonstrated that day of differentiation was a larger discriminator between samples than *NKX2-5* genotype (Fig. 2d,e; Supplementary Table 3). The canonical cardiac myomiRs, such as *MIR1-1*, *MIR126*, *MIR133* and *MIR208*, were unperturbed in *NKX2-5* knockout cardiomyocytes (Fig. 2b,e,f,g). Further, even miRNAs with proximal NKX2-5 binding sites^19^, such as *MIR138-*2 were not dysregulated (Fig. 2g). Indeed, we did not identify any miRNAs that required NKX2-5 activity in day 10 cardiomyocytes (Fig. 2e,g) for appropriate expression.

## Discussion

Using differentiating human pluripotent stem cells as a model we describe the miRNAome of pre-contractile cardiac progenitor cells and contracting cardiomyocytes. Emerging *NKX2-5* positive cardiac progenitors are capable of giving rise to three cell lineages, namely cardiac muscle, endothelial and smooth muscle cells^18,23,34^. The microRNA profile of these cardiac progenitors is similar to NKX2-5 negative mesoderm, which likely reflects the recent separation (less than 24 hours) of these lineages. However, the canonical cardiac miRNAs (referred to as the myomiRs,^9^) are upregulated in the NKX2-5 positive day 6 population, consistent with the eventual differentiation of these cells toward cardiomyocytes. By day 10 of differentiation the cardiomyocytes had upregulated 112 microRNAs, many of which had been previously identified as key drivers of myocardial function in the mouse^1,9^. This increase may be a reflection of phenotypic complexity occurring as cardiac progenitors mature into functional cardiomyocytes and require the on-going regulation of the definitive cardiomyocyte gene expression program.

A highly conserved network of transcription factors drives both cell specification and spatio-temporal patterning during heart development^35,36^. At present, our understanding of the regulatory relationships between cardiac transcription factors and the cardiac miRNAome is limited, though systematic profiling provides a platform to further define these linkages^4,37^. Given its vital role in heart development^38^, we hypothesized that the transcription factor *NKX2-5* was necessary for the regulation of the cardiomyogenic miRNAs. Here we searched for *NKX2-5* dependent miRNAs and, surprisingly, found that no miRNAs were differentially expressed between *NKX2-5* heterozyogote and null cardiomyocytes. For example, in immature human cardiomyocytes *in vitro, NKX2-5* is not required to up-regulate microRNAs, such as miR-1-2^39^, that have been identified to play important roles in mouse heart development. Therefore, the phenotype observed in *NKX2-5* null cultures is not due to perturbed miRNA expression but rather the disruption of the cardiac myogenic transcriptional network, including *HEY2*^19^. Thus, it is possible that the key developmental transcriptional networks driving cardiomyogenesis do not directly establish the miRNAome and that early stage heart development can tolerate miRNA dysregulation^5^.

A conditional knockout of Dicer, which results in a blockade of miRNA production, using *Nkx2-5* as a driver of Cre recombinase resulted in embryonic lethality at 12.5 days post-coitum (dpc), with the embryos showing pericardial edema and poorly developed ventricular myocardium^39^. However, the early stages of cardiomyogenesis were not disrupted in *Nkx2-5* driven *Dicer* knockouts with embryos at 11 dpc having normal appearance^39^. These findings suggest that cardiac microRNAs have restricted, non-critical roles early in heart development, in contrast to the essential roles that transcription factors within the cardiac gene regulatory network play. Furthermore, multiple studies implicate cardiac miRNAs in the pathophysiology of stress responsive cardiac hypertrophy^40–44^. Taken together these data suggest miRNAs have key roles in cardiomyocyte function and physiology, possibly as regulators of stress-responsive gene expression programs, rather than development. In this context, human pluripotent stem cell models provide an avenue to investigate the role of miRNAs in the pathogenesis of human myocardial disease and assess efficacy of miRNA-based therapeutics in a pre-clinical setting.

A caveat to this study is that differentiating human pluripotent stem cells lack the precise spatio-temporal cues present in the embryo. Therefore, it may be that the miRNA sequence profiles presented here do not fully reflect those found in the developing human foetus. In addition, this study focuses on very early stages of cardiogenesis and the regulatory role of *NKX2-5* may change over developmental time. A second limitation is that miRNA expression may be perturbed in NKX2-5^eGFP/w^ cardiomyocytes, however, we have previously demonstrated that NKX2-5 heterozygosity does not impact cardiomyocyte differentiation at this early stage^18,19^. It is possible that later in cardiomyocyte development *NKX2-5* is important for maintaining the appropriate mix of miRNAs in the heart. Nevertheless, our data suggest that in the early stages of human heart development the miRNAome does not require *NKX2-5*. Conversely, other key regulators of cardiac muscle development such as MEF2c, TBX5 and GATA4^2–4,45,46^, which are not disrupted in *NKX2-5* null cardiomyocytes^19^, may have a role in regulating cardiomyogenic miRNAs. Studies focussed on the regulatory nexus between the cardiac gene regulatory network^19,47,48^ and the miRNAome^17^ in the human context will be required to fully understand the transcriptional regulation of the cardiac microRNA network.

## Methods

### Ethical approvals

All experiments were approved by the Royal Children’s Hospital Research Ethics Committee (HREC 33001 A). Methods were carried out in accordance with the relevant guidelines and regulations provided by National Health and Medical Research Council (National Statement on Ethical Conduct in Human Research).

### Cell culture and cardiac differentiation

All cell culture reagents were purchased from Thermo Fisher unless stated otherwise. HES3 *NKX2-5*^*eGFP/w*^ and *NKX2-5*^*eGFP/eGFP*^ cell lines were routinely passaged using TrypLE Select and maintained on tissue culture flasks, as previously described^19^. To induce differentiation, hESCs were dissociated into single-cell suspension using TrypLE Select and seeded onto Geltrex coated (1:100 dilution) culture plates at 2.5 × 10^5^ cells/cm2 in basal differentiation media consisting of RPMI (Thermo 61870), B27 minus vitamin A (Thermo 12587) and 50 µg/ml ascorbic acid (Sigma A92902), further supplemented with 10 µM CHIR99021 (Tocris Bioscience 4423) and 80 ng/mL Activin A (Peprotech). Following 24 and 96 h of induction, media was replaced with basal media supplemented with 5 µM IWR-1 (Sigma I0161) and from day 5 onwards, differentiating cultures were maintained in basal media only until harvested for analysis.

### FACS analysis

To isolate cardiac-cell lineages from non-cardiac cell lineages, live cell sorting based on eGFP expression of differentiating cultures at either day 6 or day 10 were performed. Differentiating hESCs were dissociated, filtered through a 40 µM cell strainer and resuspended in PBS containing 2% fetal calf serum and 1 µg/mL propidium iodide. Flow cytometric gates of GFP positive and negative cells were set using negative control HESCs cultured in basal media containing 100 ng/ml bFGF. Sorted GFP positive and negative cells on day 6 and day 10 of differentiation were collected and snap frozen prior to RNA extraction. Cell sorting was done using BD Influx^TM^ cell sorters (BD Biosciences) and flow cytometric data was analyzed using Flowlogic software (Inivai Scientific).

### Next generation sequencing and bioinformatics analysis

Total RNA from GFP sorted populations on day 6 and day 10 differentiating cultures were prepared in triplicate using the miRNeasy Mini (Qiagen 217004). A minimum amount of 500 ng of total RNA was analyzed for RNA integrity and submitted for sequencing. Small RNA libraries were prepared using Truseq Small RNA Sample Prep Kit (Illumina) and small RNA sequencing was performed on the Illumina Hi-Seq platform using the Illumina CASAVA 1.8.2 software (Australian Genomic Research Facility). Short RNAs were sequenced as 50 base pair single end reads and all adapter and primer sequences were removed using *cutadapt*^49^. Bowtie aligner was used to map the 17-26 base pair single end reads to the human reference genome (hg19) allowing one mismatch. The uniquely mapped reads were summarized across microRNAs with featureCounts (Rsubread v1.20.6)^50^ using ensemble miRNA gene annotation. Lowly expressed miRNAs were filtered out (less than 10 counts per million in fewer than three samples). The data was Voom transformed with cyclic loess normalization, and differential expression assessed using empirical Bayes moderated t-tests from the R Bioconductor limma package (Version 3.20.9)^51^ through the statistical language R. Unbiased hierarchical clustering was performed using standard complete linkage and Euclidean distance. Data has been deposited on GEO (GSE134852).

### Quantitative PCR

To analyze miRNA gene expression levels, quantitative real time PCR was performed on differentiating cultures at day 6 and day 10. 1 µg of RNA was reversed transcribed using SuperScript® III (Invitrogen) and miRNA expression levels were determined using Taqman Gene Expression Assays with Taqman Universal PCR Master Mix (ThermoFisher) on the ABI 7300 Real-Time PCR detection system (Applied Biosystems). Transcript expression levels were normalized to the averaged expression of the reference gene snoRNA *RNU24*, and gene relative quantification was calculated using the 2^−∆∆**Ct**^ method.

## Supplementary information

Supplementary Information

Supplementary Table 2

Supplementary Table 3

## Data Availability

The authors declare that all data supporting the findings of this study are available within the article and its supplementary information files or the GEO database (http://www.ncbi.nlm.nih.gov/geo/, accession codes GSE134852) or from the corresponding author (DAE) upon reasonable request.
