# Publisher Correction: The role of cardiac transcription factor NKX2-5 in regulating the human cardiac miRNAome

**DOI:** 10.1038/s41598-019-55970-6

**Published:** 2019-12-27

**Authors:** Deevina Arasaratnam, Katrina M. Bell, Choon Boon Sim, Kathy Koutsis, David J. Anderson, Elizabeth L. Qian, Edouard G. Stanley, Andrew G. Elefanty, Michael M. Cheung, Alicia Oshlack, Anthony J. White, Charbel Abi Khalil, James E. Hudson, Enzo R. Porrello, David A. Elliott

**Affiliations:** 1 Murdoch Children’s Research Institute, Royal Children’s Hospital, Flemington Road, Parkville, Victoria, 3052 Australia; 2 Australian Regenerative Medicine Institute, Monash University, Clayton, Victoria, 3800 Australia; 3 Department of Pediatrics, The Royal Children’s Hospital, University of Melbourne, Parkville, Victoria, 3052 Australia; 4 Department of Anatomy and Developmental Biology, Monash University, Clayton, Victoria, 3800 Australia; 5 Monash Heart, Monash Medical Centre, Monash University, Clayton, Victoria, 3800 Australia; 6 Department of Genetic Medicine and Medicine, Weill Cornell Medical College-Qatar, Doha, Qatar; 7 QIMR Berghofer Medical Research Institute, Herston, Queensland 4006 Australia; 8 Department of Physiology, School of Biomedical Sciences, The University of Melbourne, Parkville, Victoria, 3010 Australia

Correction to: *Scientific Reports* 10.1038/s41598-019-52280-9, published online 04 November 2019

In the original version of this Article, the author Charbel Abi Khalil was incorrectly indexed. This error has now been corrected.

