# Supplementary material for: The role of cardiac transcription factor NKX2-5 in regulating the human cardiac miRNAome
Source: Sci Rep. 2019 Nov 4;9:15928. doi: 10.1038/s41598-019-52280-9 (PMC6828809; doi:10.1038/s41598-019-52280-9)
Supplement: Supplementary file 1 — Supplementary Information [file 41598_2019_52280_MOESM1_ESM.pdf]

Supplementary Information

The role of cardiac transcription factor *NKX2-5* in regulating the human cardiac miRNAome.

Deevina Arasaratnam[1,2], Katrina M. Bell[1], Choon Boon Sim[1], Kathy Koutsis[1], David J. Anderson[1], Elizabeth L. Qian[1], Edouard G. Stanley[1,3,4], Andrew G. Elefanty[1,3,4], Michael M. Cheung[1,3], Alicia Oshlack[1], Anthony J. White[5], Charbel Abi Khalil[6], James E. Hudson[7], Enzo R. Porrello[1,8], and David A. Elliott[1,2,3]*.

Supplementary Information

Supplementary Table 1. Summary of small RNA sequencing data.

Sample	miRNA reads	% reads miRNA	snoRNA reads	% reads snoRNAs
d6 GFP^{neg}	2061961	22	403256	4.3
d6 GFP^{neg}	1814338	25	124178	1.7
d6 GFP^{neg}	2141959	22	431677	4.4
d6 GFP^{+}	1822388	24.5	138515	1.9
d6 GFP^{+}	1550786	23.5	91198	1.4
d6 GFP^{+}	154552	21.9	124484	1.7
d10 GFP^{+}	2271422	27.5	141034	1.7
d10 GFP^{+}	2521212	36.8	173173	2.5
d10 GFP^{+}	2272235	30.6	166257	2.2

Supplementary Figures.

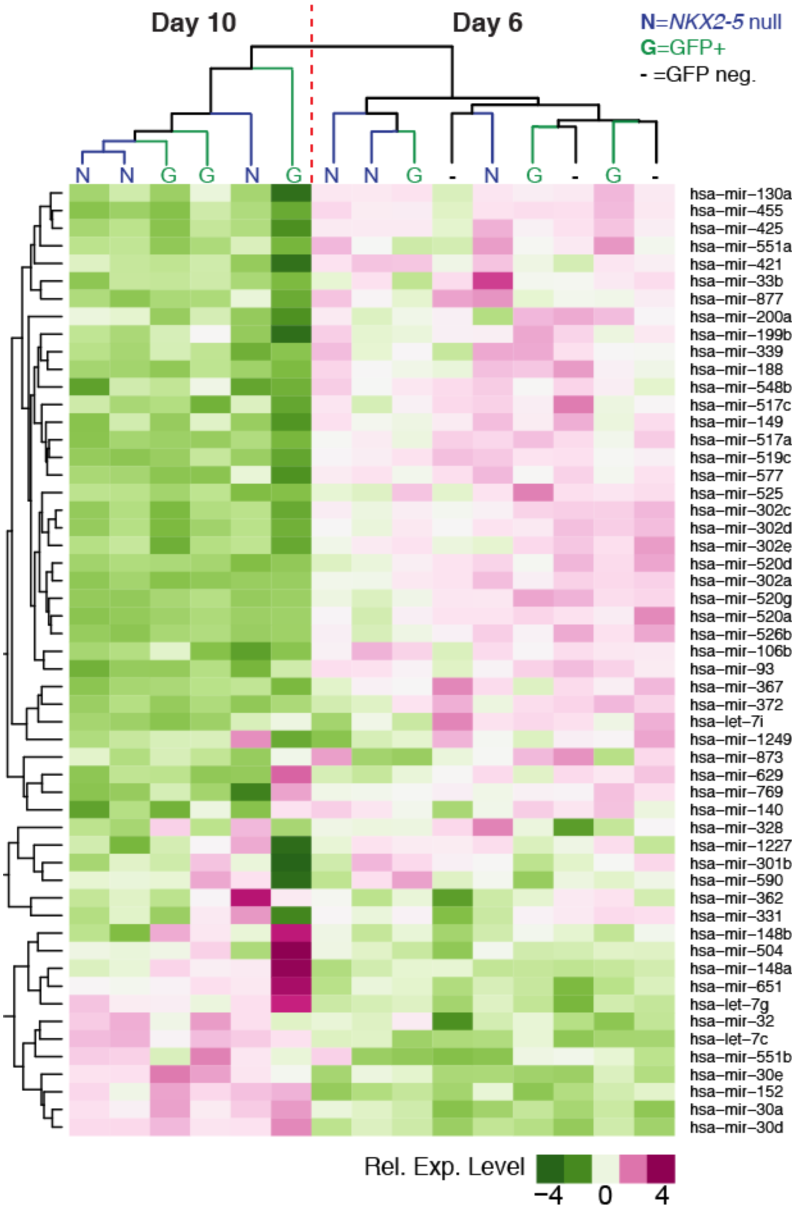

Supplementary Figure 1. Day 6 NKX2-5 positive cells are enriched for microRNAs associated with cell proliferation. Heat map of unsupervised hierarchical clustering of microRNA sequence profile from day 6 and day 10 cells based on microRNA associated with cell proliferation (log_2 RPM values). Samples cluster more closely according to day of differentiation than *NKX2-5* genotype. Differential microRNA profiles suggest the day 6 samples are more proliferative than day 10. NKX2-5 knockout cells are indicated in blue, while the *NKX2-5*^{eGFP} positive cells are shown in green and negative cells in black.

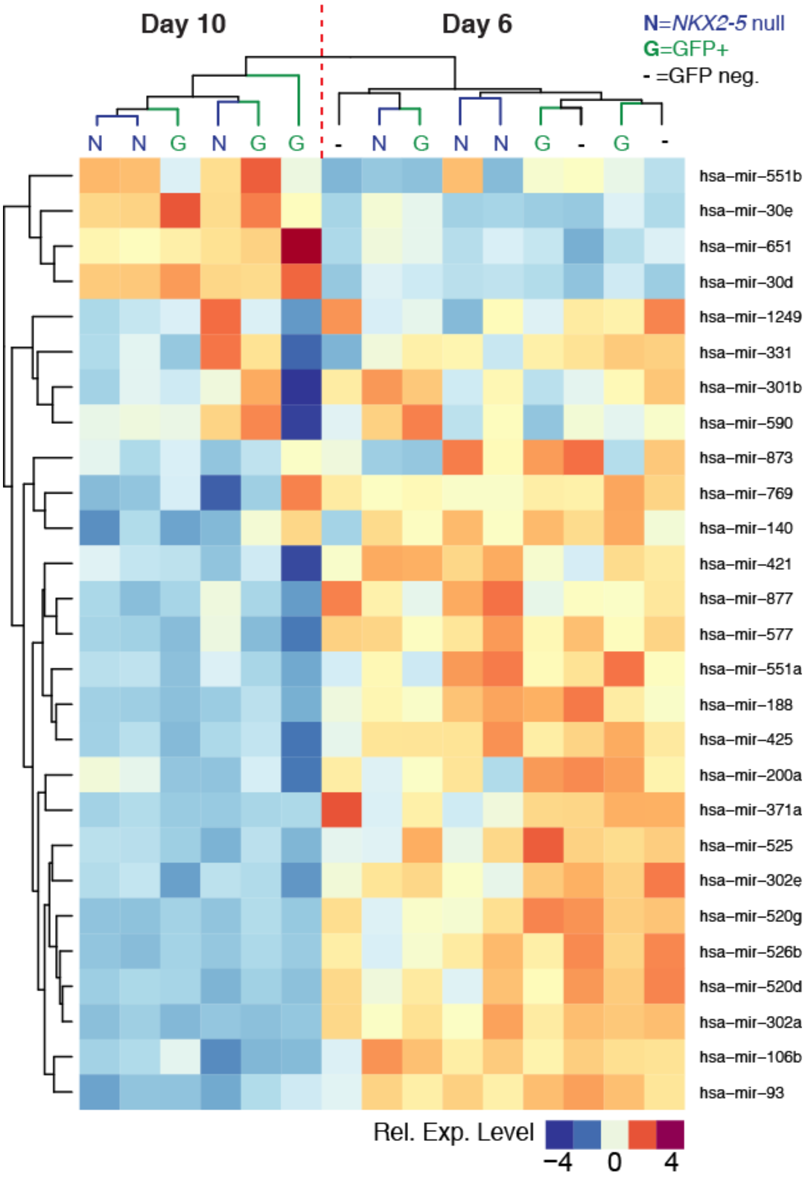

Supplementary Figure 2. Differential expression of miRNAs in the Hippo/YAP pathway during cardiac differentiation. Heat map of unsupervised hierarchical clustering of day 6 and day 10 samples based on microRNAs involved in the Hippo/YAP pathway (log_2 RPM values). Samples cluster more closely according to day of differentiation than *NKX2-5* genotype. Differential microRNA profiles suggest the day 6 samples have higher levels of Hippo/YAP signalling. NKX2-5 knockout cells are indicated in blue, while the *NKX2-5*^{eGFP} positive cells are shown in green and negative cells in black.
